# Highly Multiplexed Immunohistochemical MALDI-MS Imaging of Biomarkers in Tissues

**DOI:** 10.1021/jasms.0c00473

**Published:** 2021-02-25

**Authors:** Gargey Yagnik, Ziying Liu, Kenneth J. Rothschild, Mark J. Lim

**Affiliations:** † AmberGen, Inc., 313 Pleasant Street, Watertown, Massachusetts 02472, United States; ‡ Molecular Biophysics Laboratory, Department of Physics and Photonics Center, Boston University, Boston, Massachusetts 02215, United States

**Keywords:** mass spectrometry, matrix-assisted laser desorption/ionization, mass spectrometric imaging, photocleavable, mass-tags, bead-arrays, *in situ* hybridization, immunohistochemistry, immunofluorescence, pathology, tissue diagnostics, multiplexing

## Abstract

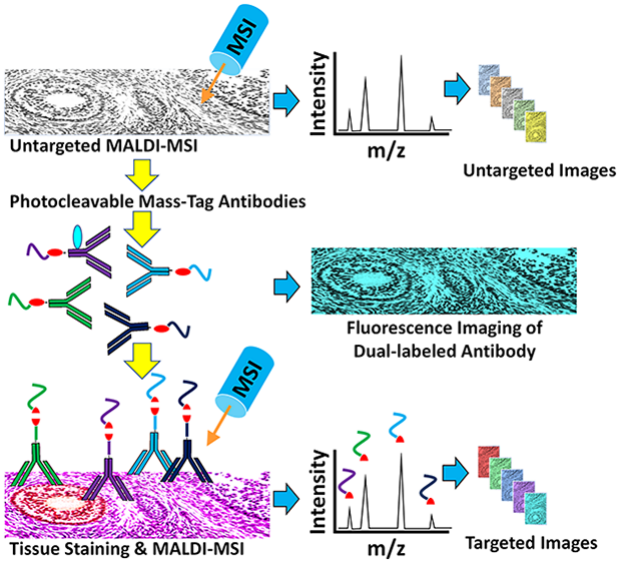

Immunohistochemistry (IHC) combined with fluorescence microscopy provides an important and widely used tool for researchers and pathologists to image multiple biomarkers in tissue specimens. However, multiplex IHC using standard fluorescence microscopy is generally limited to 3–5 different biomarkers, with hyperspectral or multispectral methods limited to 8. We report the development of a new technology based on novel photocleavable mass-tags (PC-MTs) for facile antibody labeling, which enables highly multiplexed IHC based on MALDI mass spectrometric imaging (MALDI-IHC). This approach significantly exceeds the multiplexity of both fluorescence- and previous cleavable mass-tag-based methods. Up to 12-plex MALDI-IHC was demonstrated on mouse brain, human tonsil, and breast cancer tissues specimens, reflecting the known molecular composition, anatomy, and pathology of the targeted biomarkers. Novel dual-labeled fluorescent PC-MT antibodies and label-free small-molecule mass spectrometric imaging greatly extend the capability of this new approach. MALDI-IHC shows promise for use in the fields of tissue pathology, tissue diagnostics, therapeutics, and precision medicine.

## Introduction

Immunohistochemistry (IHC) is widely used to determine the structural organization of biomolecules at the tissue, cellular, and subcellular levels.^1−3^ For example, IHC is the preferred method for studying extracellular amyloid plaques and intracellular Tau-based neurofibrillary tangles in neurodegenerative disorders.^4,5^ In oncology, IHC can be used to diagnose, classify into subtypes, and determine the optimal treatment of various cancers,^6,7^ including the evaluation of tumor-infiltrating lymphocytes (TILs) that are of prognostic value.^8^ IHC analyses are generally performed on tissue samples, for example, those collected by biopsy or the surgical resection of a tumor. Typically, tissue samples are fresh-frozen or formalin-fixed and paraffin-embedded (FFPE), then thin-sectioned (e.g., 3–10 μm) and mounted on glass microscope slides. Fluorophores or chromogenic agents conjugated to antibody probes are the most common method of visualizing the spatial distribution of targeted biomolecules using microscopy.^3^

It is often important to simultaneously determine the localization and potential colocalization of a number of biomarkers. For example, this is critical to map the location of the hundreds of possible proteins involved in cell regulation and dysregulation in a highly heterogeneous tissue.^9,10^ However, fluorescence microscopy is limited to the simultaneous detection of only a few biomarkers, since molecular fluorophores exhibit relatively broad excitation and emission bands that result in spectral overlap.^2^ The multiplexing limit of standard fluorescence microscopy is generally 3–5, while hyperspectral and multispectral methods are limited to 8.^2,11−13^ Furthermore, these multiplexing methods often require cycling strategies (e.g., PerkinElmer’s OPAL multispectral platform, t-CyCIF,^14^ and CODEX^15^) such as iterative staining followed by photobleaching or probe removal and denaturation.^9,16−18^ Such methods are complex and laborious, and incomplete cycling can confound the results.^9,19^

In contrast, mass spectrometric imaging (MSI) facilitates a high level of multiplexing without the limitations of the aforementioned optical methods (limited only by the mass resolution, which is typically less than 1 Da). Briefly, these methods scan the tissue specimen with a mass spectrometer and generate a full mass spectrum at each “pixel”, thereby allowing the simultaneous imaging of any given mass species within the spectra.^20^ The Caprioli group first introduced this technique based on MALDI-MS,^21^ which has since been widely adopted for the direct label-free imaging of biomolecules, including proteins, nucleic acids, lipids, metabolites, and even small drug compounds in complex tissues.^22^ This technique has also been extended to other mass spectrometry (MS) approaches, such as ESI-based DESI-MS imaging.^23^ While MALDI and DESI MSI approaches do not currently match the 0.2 μm spatial resolution of optical methods (e.g., the 10 μm laser focus with the newer Bruker rapifleX MALDI-MS instruments), it is possible to obtain an improved resolution using innovative designs such as transmission geometry (2 μm)^24^ or atmospheric-pressure MALDI-MSI with laser focusing objectives (1.4 μm).^25^

However, the MSI of intact macromolecules such as proteins is typically not possible due to the insufficient mass resolution and poor sensitivity.^22^ Identification of a particular biomolecule requires tandem MS/MS fragmentation, ultrahigh mass resolution instruments, and bottom-up proteomic approaches (e.g., the *in situ* proteolysis of the tissue). To overcome this limitation, several targeted MSI approaches have been introduced that allow multiplex workflows similar to those of conventional IHC and *in situ* hybridization (ISH) using labeled antibody and nucleic acid probes. TAMSIM (targeted multiplex mass spectrometric imaging) is a matrix-free laser desorption/ionization (LDI) method, which uses antibodies conjugated to small organic photocleavable mass-tags that are cleaved and ionized during MSI.^26^ However, the mass-tags are not readily synthesized, and only three-plex imaging has been shown thus far.^27^

In contrast, peptide mass-tags are easily produced using standard solid-phase synthesis, the masses are readily tuned by altering the sequence, and peptides generally ionize with a high efficiency. Lemaire et al. first introduced a photocleavable peptide-based MALDI-MSI method for the targeted imaging of tissues, termed Tag-Mass.^28^ However, the mass-tagging of the probe (e.g., antibody) is a complex multistep process involving an intermediate chemical linker. These drawbacks have thus far limited the general utilization of Tag-Mass, and consequently only two-plex MSI has been achieved to date.^28−30^

Imaging mass cytometry uses antibodies tagged with rare-earth metals combined with inductively coupled plasma mass spectrometry (ICP-MS),^19^ and multiplexed ion beam imaging by time-of-flight (MIBI-TOF)^31^ uses antibodies tagged with metal isotopes combined with secondary ion mass spectrometry (SIMS) imaging. This subcellular resolution approach has achieved the highest multiplexing level to date with at least 32-plex tissue staining (e.g., imaging mass cytometry). However, this method is suitable only for scanning small areas of a tissue specimen (e.g., 1 mm^2^) at subcellular resolution (1 μm) due to the long scan times required. In addition, this method requires specialized MS instrumentation and is a destructive approach that reduces compounds to elements and is therefore not compatible with performing an untargeted direct MSI analysis of biomolecules (in conjunction with the targeted MSI using mass-tagged probes). Furthermore, the probe labeling process is also highly complex, involving preloading a polymer with a metal ion, partially reducing the antibody, and coupling the polymer and antibody together with multiple purifications of the polymer and antibody.^32^

Here we report a new method for combining MALDI-MSI with IHC (termed MALDI-IHC) based on the development of novel photocleavable mass-tags (PC-MTs) and associated sample preparation methods, which overcome the aforementioned limitations. PC-MTs are modified polypeptides comprised of a mass reporter region, a high-efficiency photocleavable linker (PC-Linker) incorporated into the peptide through the solid-phase synthesis, and an *N*-hydroxysuccinimide (NHS)-ester probe-reactive moiety near the C-terminal. PC-MT antibody probes are produced in a one-step reaction. The fast and efficient photonucleus^33^ used in the novel PC-Linker provides a robust sensitivity in practice, allowing the high-plex MSI of a wide range of biomarkers in a variety of tissues, including mouse brain, human tonsil, and breast cancer, as shown here. Furthermore, novel dual-labeled antibodies combining both PC-MTs and fluorophores allowed the direct correlation of MSI with conventional immunofluorescence. Finally, the versatility of the approach is shown through the ability to perform both label-free untargeted small-molecule MSI and multiplex PC-MT-based targeted MSI of macromolecular biomarkers on the same tissue section. Label-free untargeted small-molecule MSI can directly analyze lipids, drugs, and metabolites, which is not possible using standard IHC or imaging mass cytometry.

## Methods

### Materials

See the Supporting Information. Methods for PC-MT and PC-MT-antibody preparation as well as methods for MALDI-MSI are provided here; see the Supporting Information for multiplex mass spectrometry-based immunohistochemistry (MALDI-IHC), bead-array procedures, and immunofluorescence staining.

#### Photocleavable Mass-Tags (PC-MTs)

**Figure 1:**
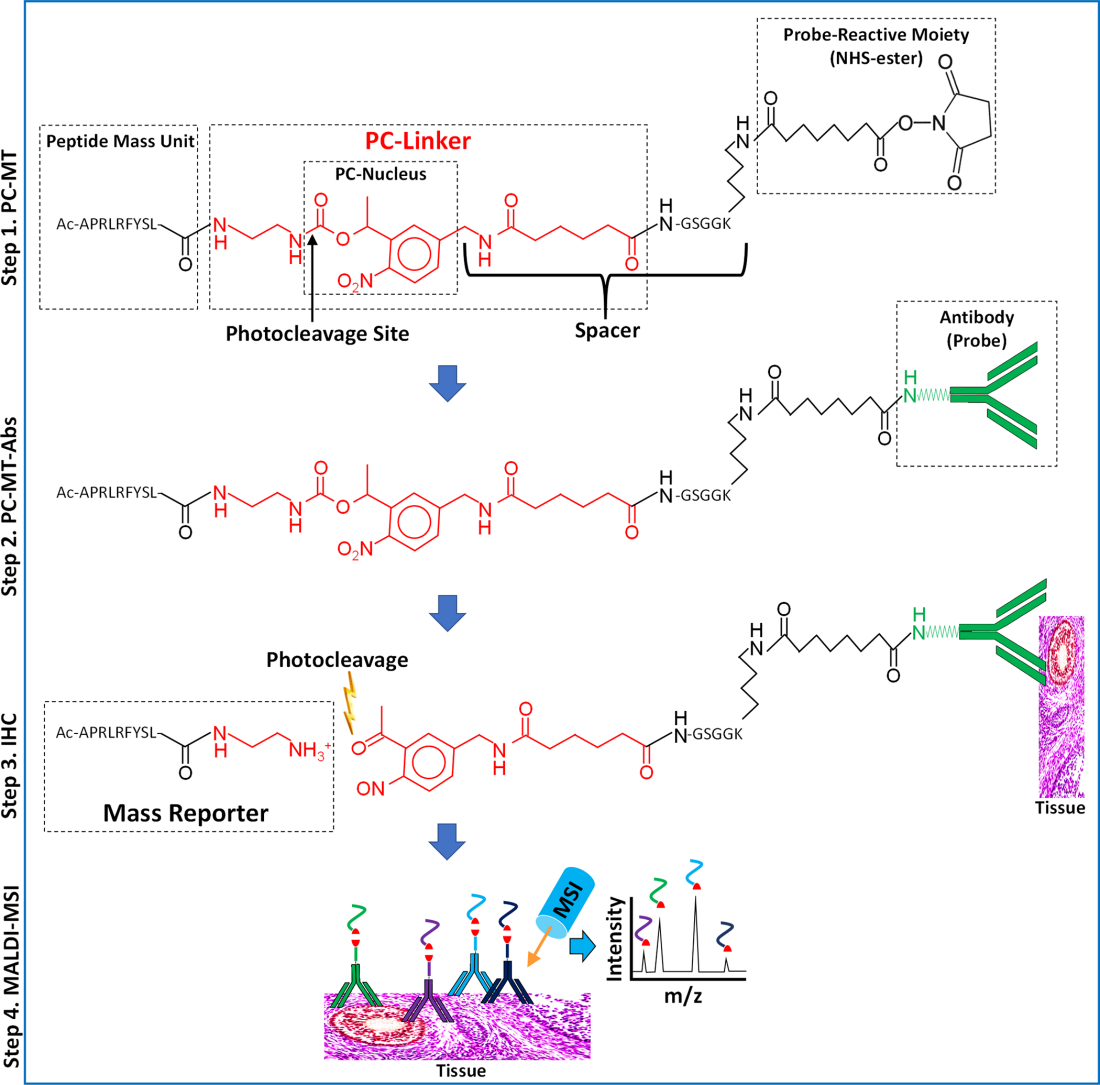
Structure and use of PC-MTs and PC-MT antibodies (PC-MT-antibodies). (Step 1, PC-MT) The structure of a PC-MT is shown. To produce the PC-MTs, an amine-terminal Fmoc-protected version of the photocleavable linker (PC-Linker; red) is incorporated during conventional Fmoc-based solid-phase peptide synthesis (SPPS) along with the other amino acids. The example PC-MT shown is oriented with the N-terminal on the left and the C-terminal on the right. “APRLRFYSL” is an example amino acid sequence of the peptide mass unit (see Supplementary Table S1 for all mass units used in this work). The PC-MTs contain a spacer that connects the 1-(2-nitrophenyl)-ethyl-based photocleavable nucleus (PC-Nucleus) to the probe-reactive moiety. This spacer is comprised of a portion of the PC-Linker plus the GSGGK amino acid sequence. The probe-reactive moiety (an NHS-ester leaving group) is generated during SPPS on the ε-amine of the lysine (K) included in the spacer. N-Terminal acetylation (“Ac”) of the α-amine is used to prevent the self-reaction and polymerization of the PC-MT. (Step 2, PC-MT-antibodies) The NHS-ester probe-reactive moiety of the PC-MTs is then reacted in one step with native primary amines in the antibody probes to form the PC-MT antibodies (PC-MT-antibodies). (Step 3, IHC) Immunohistochemistry (IHC) is performed by binding the PC-MT-antibodies to targets in thin tissue sections mounted on conductive microscope slides, followed by photocleavage under dry conditions to liberate the mass reporters. Note that a small residual portion of the PC-Linker (red) remains as part of the photocleaved mass reporters. (Step 4, MALDI-MSI) Finally, MALDI mass spectrometric imaging (MSI) is performed following the application of a matrix compound (matrix not shown). The inverted Y-shaped structures are antibodies, and the curved colored lines correspond to the mass units from different PC-MTs. The red shapes correspond to the photocleaved PC-Linker.

The N-terminal acetylated and NHS-ester-activated peptide-based PC-MTs (Figure 1, step 1) were produced commercially by standard solid-phase peptide synthesis (SPPS).^34^ An Fmoc-protected version of the photocleavable linker (PC-Linker) shown in Figure 1 was incorporated in the same manner as the amino acids. To create the probe-reactive moiety on the PC-MTs, conversion of the ε-amine of the lysine (K) in the spacer to an NHS-ester was achieved using disuccinimidyl suberate (DSS). The use of bifunctional succinimidyl esters such as DSS or DSC (disuccinimidyl carbonate) has been previously reported for conversion of primary amines to NHS-esters.^35^

#### Preparation of PC-MT Antibodies (PC-MT-Antibodies)

The antibody (100 μL,1 μg/μL in PBS) was supplemented with a 1/9th volume of 1 M sodium bicarbonate, followed by the addition of a sufficient amount of PC-MT from a 1 mM stock in anhydrous DMF for a 10-fold molar excess relative to the antibody. Reactions were carried out for 1 h with gentle mixing. In some cases for dual-labeling with a PC-MT and fluorescence, DyLight 650 NHS-ester was next added from a 5 mM stock prepared in anhydrous DMF for a 15-fold molar excess relative to the antibody (reacted for 30 min more with gentle mixing). Reactions were then quenched by a 1/9th volume of 1 M glycine, followed by mixing for 15 min. Finally, a 1/199th volume of a 10% (w/v) BSA carrier stock in water was added for a final amount of 0.05% BSA (w/v). To remove the unreacted labeling reagent, PC-MT-antibodies were processed on PD SpinTrap G-25 columns according to the manufacturer’s instructions, using TBS (50 mM Tris, pH 7.5, 200 mM NaCl) as the pre-equilibration buffer. The resultant PC-MT-antibodies were further supplemented with a 1/9th volume of 10× TBS.

#### Photocleavage, Matrix Application, and MALDI-MSI

Following PC-MT-antibody probing procedures (see the Supporting Information and Methods), dried bead-array substrates or tissue slides were illuminated for 5 min with 365 nm light (LED Cube 100 IC from Honle UV Technology, Marlboro, MA). Next, the matrices DHB (for PC-MTs) or DAN (for direct lipid analyses) were applied to the bead-array substrates or tissue slides by sublimation, followed by recrystallization according to published reports.^36,37^ MALDI-MSI was achieved with a rapifleX MALDI-TOF-MS instrument (Bruker Daltonics, Billerica, MA) using the following parameters: reflector mode (the positive ion mode for PC-MTs and the negative ion mode for direct lipid analyses); laser spot size of 10 or 20 μm with 10 or 20 μm continuous raster scanning, respectively; 300–500 laser shots per pixel; 30–50% typical laser power setting; and normalization to the total ion count (TIC) for the tissue PC-MT images. Image and spectral analysis were performed using flexImaging and flexAnalysis software (Bruker Daltonics, Billerica, MA).

## Results and Discussion

### Design and Synthesis of Novel PC-MTs and PC-MT Probes

The structure of the peptide-based PC-MT and steps used to produce a PC-MT-antibody for tissue MALDI-MSI are shown in Figure 1. The PC-MTs are comprised of an NHS-ester probe-reactive moiety near the C-terminal, a peptide mass unit on the N-terminal, and a photocleavable linker (PC-Linker) in between. The PC-Linker is comprised of a fast and efficient 1-(2-nitrophenyl)-ethyl-based photocleavable nucleus^33^ (PC-Nucleus), with the photocleavage site indicated in Figure 1, step 1. Positioned between the PC-Nucleus and the probe-reactive moiety is a spacer comprised of a portion of the PC-Linker and a GSGGK amino acid sequence. Finally, the N-terminal α-amine of the mass unit was acetylated to prevent the self-reaction or polymerization of the PC-MT labeling reagent. While Figure 1 shows an example mass unit, 29 different PC-MTs were made for this work, each with unique mass unit masses that were achieved using different amino acid sequences or through the use of stable isotopic amino acids (see Supplementary Table S1 and Supplementary Table S1.1). The monoisotopic masses of the photocleaved mass reporters are also listed in Supplementary Table S1, which includes the N-terminal acetylation, the mass unit, and a small portion of the photocleaved PC-Linker as shown in Figure 1, step 3.

To produce a PC-MT-probe, the NHS-ester probe-reactive moiety of the PC-MT reagent was reacted with an antibody (Figure 1, step 2). This was accomplished in one step, followed by size-exclusion chromatography using micro-spin columns to remove the unreacted reagent. This antibody-labeling reaction is substantially simpler than the multistep method previously reported by Lemaire et al.^28^ In that case, the antibody is first conjugated to the non-photocleavable heterobifunctional cross-linker MBS (3-maleimidobenzoic acid *N*-hydroxysuccinimide ester) by a reaction with its NHS-ester moiety. The antibody is then purified by size-exclusion chromatography (to remove the unreacted MBS linker). The sulfhydryl-reactive maleimide moiety generated on the antibody by the attached MBS linker is then reacted with a photocleavable peptide containing a terminal cysteine. The final labeled antibody is again purified, this time by dialysis. In contrast, the single-step conjugation process facilitated by our improved PC-MT reagent not only provides a simpler method to produce PC-MT-probes but can also result in an increased labeling efficiency and less antibody loss due to the fewer number of processing steps.

### Highly Multiplexed MALDI-MSI using PC-MT-Antibodies on Random Bead-Arrays

To demonstrate multiplexing with PC-MT antibody probes (PC-MT-antibody), we first used an approach based on photocleavable bead-array mass spectrometry (PC-BAMS),^38,39^ which involves depositing microbeads in a random array on substrates formatted in the footprint of a microscope slide that can be scanned by MALDI-MSI. Fifteen different PC-MT versions of an anti-streptavidin antibody were created and used *separately* to probe 20 μm polymer streptavidin beads (see Supplementary Table S1 for PC-MT assignments to the streptavidin antibodies and mass reporter masses). Following probing and washing, the 15 bead species were pooled and deposited as a random array on indium tin oxide (ITO)-coated microwell slides. The dried arrays were then illuminated with UV light to photocleave the PC-MTs, the MALDI matrix was applied by sublimation and recrystallization, and the array was imaged by MALDI-MSI in the positive ion reflector mode. Supplementary Figure S1 shows a colorized MALDI-MS image of the bead-array (i.e., an *xy*-intensity map of the different *m*/*z* values). The different colors in the inset image correspond to the different monoisotopic *m*/*z* values for a particular mass reporter from a particular PC-MT. Fifteen different spatially resolvable 20 μm bead species (and many replicates of each) were observed, as identified by their unique mass reporters derived from the bound PC-MT-antibodies. Color-coded overlaid MALDI-MSI spectra from single pixels from representative single beads within the array show the mass reporter peaks (Supplementary Figure S1, black arrows), with no bead-to-bead “cross-talk” observed. For a basic assessment of the PC-MT signal consistency across different regions of the array, the entire scanned region was divided into four equally sized quadrants, and the average spectrum was obtained from each. As an example, the monoisotopic peak intensity for the PC-MT comprised of mass unit 1 produced a % CV of 18% across the four quadrants.

This demonstrates that at least 15-plex MSI is readily achievable with these PC-MTs. Given the typical MALDI-TOF mass resolution (e.g., substantially better than 1 Da in the reflector mode) and mass accuracy (e.g., 50–100 ppm), hundreds of unique PC-MT species would be discernible in the 850–1250 *m*/*z* mass window alone, as shown in Supplementary Figure S1. Furthermore, selecting from the 20 standard natural amino acids, >100 000 octameric peptide-based PC-MTs of distinct masses could be produced.

### Multiplex MALDI Mass Spectrometry-Based Immunohistochemistry (MALDI-IHC) on FFPE Mouse Brain Tissue Specimens

**Figure 2:**
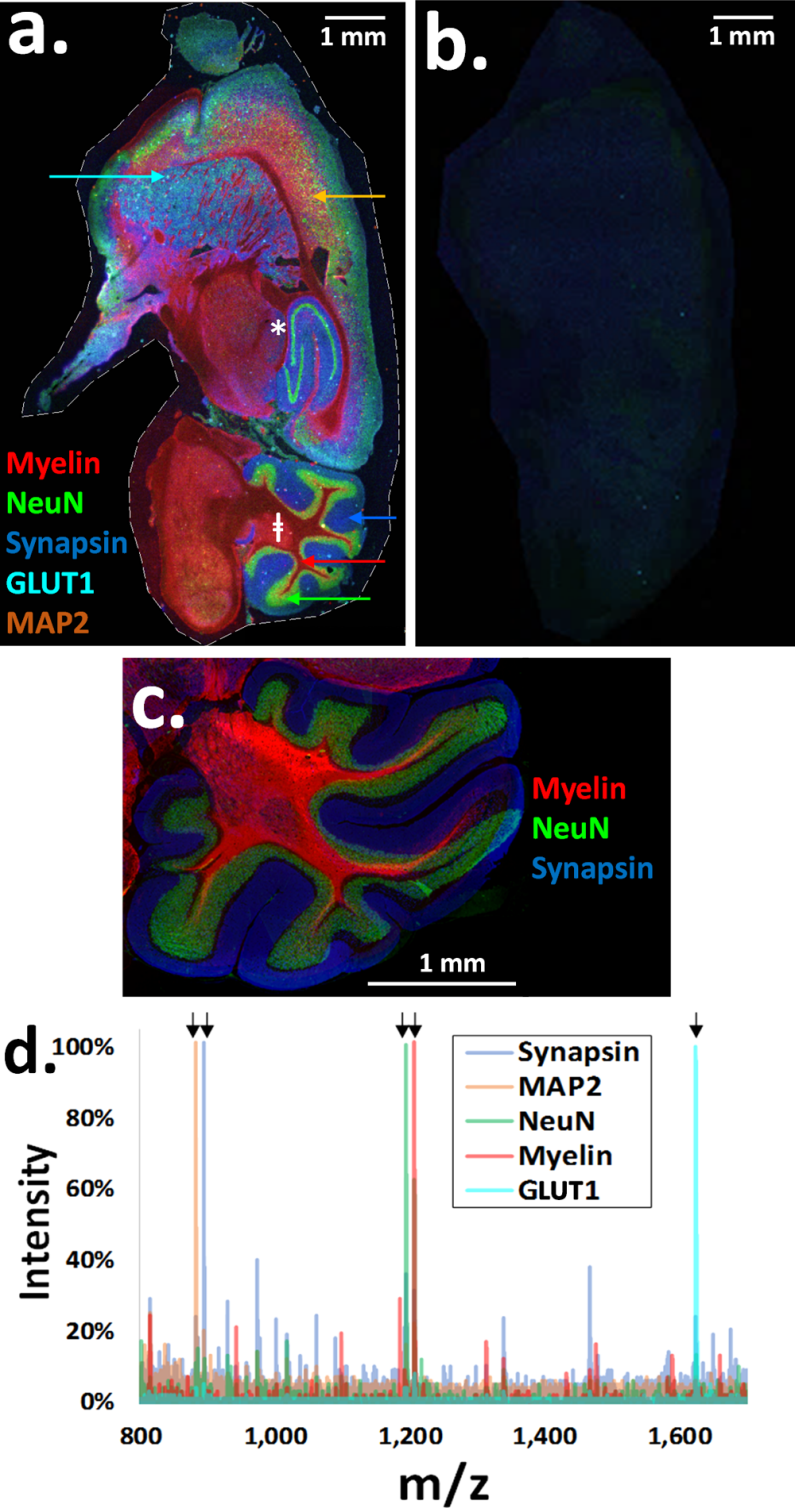
Multiplex MALDI mass spectrometry-based immunohistochemistry (MALDI-IHC) on five biomarkers in mouse-brain FFPE sagittal tissue sections. FFPE tissue sections were stained simultaneously with PC-MT-antibodies against five different biomarkers, then subjected to MALDI-MSI (in the positive ion reflector mode). Standard immunofluorescence staining was also performed on adjacent tissue sections. (a) Colorized five-plex MALDI-MS image corresponding to the monoisotopic mass reporter *m*/*z* values for the following PC-MT-antibodies (color coding is also noted on the image): myelin (red), NeuN (green), synapsin (blue), GLUT1 (cyan), and MAP2 (orange). The display scale (arbitrary peak intensity units), is as follows (minimum intensity/full intensity threshold): 0/27 (myelin), 0/12 (NeuN), 0/7 (synapsin), 0/8 (GLUT1), and 0/21 (MAP2). The hippocampus (∗) and cerebellum (⧧) were observed among other brain structures (all MALDI-MS images have a 20 μm spatial resolution). (b) MALDI-MS image from an adjacent tissue section stained with a rabbit IgG isotype control. The rabbit IgG was labeled with the same PC-MT as the rabbit polyclonal GLUT1 antibody (a mouse IgG isotype control labeled with the same PC-MT as the mouse monoclonal anti-MAP2 antibody was also tested with the same results, not shown). (c) Colorized immunofluorescence overlay of the cerebellum for synapsin (blue), NeuN (green), and myelin basic protein (red). Immunofluorescence was performed in the “single-plex” mode on adjacent tissue sections, and images were colorized and overlaid. (d) Color-coded overlaid MALDI-MS spectra are shown for selected pixels from the five-plex MALDI-MS image (the pixels are denoted with color-coded arrows in panel a). Black arrows in the spectra denote the mass reporter peaks as follows: myelin, 1206.72 *m*/*z* (red); NeuN, 1194.67 *m*/*z* (green); synapsin, 894.52 *m*/*z* (blue); GLUT1, 1624.80 *m*/*z* (cyan); and MAP2, 882.46 *m*/*z* (orange). Note that while natural isotopes of the mass reporters separated by 1 Da are easily resolved by the MALDI-MS, they are not discernible in the spectra provided due to the compact *x*-axis scaling.

Five-plex MALDI-IHC was performed on FFPE sagittal mouse brain sections. For this purpose, different PC-MTs were directly conjugated in one step to primary antibodies, as described in Figure 1. The five antibodies were targeted to myelin basic protein (a well-known axonal sheath marker),^40^ NeuN (neuronal nuclear marker),^41^ synapsin (a synaptic protein),^42^ GLUT1 (enriched in the blood capillaries of brain tissue),^43^ and MAP2 (a microtubule-associated protein present in nervous tissue).^44^Figure 2a shows the five-color MALDI-MS image of the whole brain section. The different colors correspond to the different monoisotopic *m*/*z* values for the mass reporters from the PC-MTs (see Supplementary Table S1 for PC-MT assignments for each antibody and mass reporter masses). Myelin, NeuN, and synapsin are the most prominent and produce the most distinct structural patterns. For instance, NeuN produces the distinct “swirl” pattern of the hippocampus (denoted with a “∗” in Figure 2a). Furthermore, myelin, NeuN, and synapsin highlight three distinct layers of the cerebellum (denoted with “⧧” in Figure 2a). To assess the background, PC-MT-labeled isotype controls were used to probe adjacent tissue sections at the same concentrations as their respective antibodies. For example, Figure 2b shows a MALDI-MS image of a tissue section probed with rabbit IgG carrying the same PC-MT as the rabbit polyclonal GLUT1 antibody. No significant background or distinct structural patterns were observed (PC-MT mouse IgG isotype controls, not shown, also produced no signals). It should be noted that due to the very extensive tissue processing in aqueous and organic solvents during the MALDI-IHC procedures (e.g., deparaffinization, antigen retrieval, blocking, probing, and washing), endogenous tissue-derived molecules that are not fixed in place (by the formalin fixation) are washed away and generally not detected by MALDI-MSI.

For a further demonstration of the specificity, we show that the MALDI-IHC results clearly match conventional immunofluorescence images. For example, myelin, NeuN, and synapsin antibodies directly labeled with only a fluorophore produce the same pattern in the cerebellum as that from MALDI-IHC (see Figure 2c for a colorized overlaid image of fluorescence staining in the cerebellum). Color-coded overlaid mass spectra are shown in Figure 2d for selected pixels that predominantly reflect one type of protein from the five-plex composite MALDI-MS image (those pixels marked with arrows in Figure 2a).

### Multiplex MALDI Mass Spectrometry-Based Immunohistochemistry (MALDI-IHC) on FFPE Human Tonsil and Breast Cancer Tissue Specimens

A model 12-plex antibody panel for assessing the breast cancer tumor microenvironment was constructed (see Methods in the Supporting Information). Note that the purpose of this panel was to demonstrate the applicability of the MALDI-IHC method for imaging biomarkers in cancer specimens and was not to determine the validity of particular biomarkers for the detection of any specific cell or cancer type. For this purpose, 12 biomarkers were chosen (see Supplementary Table S1). These include the breast cancer-related biomarkers^45^ estrogen receptor (ER), progesterone receptor (PR), human epidermal growth factor receptor 2 (HER2), and Ki67 (proliferation biomarker); biomarkers for tumor-infiltrating lymphocytes (TILs) and other immune-related cells,^8,9,46,47^ which included the T-cell subset biomarkers CD3 (T-cells), CD4 (T-helper), CD8 (cytotoxic T-cells), and CD45RO (memory T-cells), the B-cell biomarker CD20, and CD68, a biomarker for macrophages and other mononuclear phagocytes; a pan-cytokeratin (CK) antibody as a general epithelial cell biomarker;^48^ and finally a histone H2A.X antibody as a nuclear biomarker.^49^

Each antibody was directly labeled with a unique PC-MT (see Supplementary Table S1 for PC-MT assignments for each antibody and mass reporter masses). Notably, to eliminate a bias from the variable MALDI-MS ionization efficiencies that would occur with different PC-MT amino acid sequences, eight of the PC-MTs were comprised of either mass unit 1 (see Supplementary Table S1 for all mass units) or the same sequence comprised of various stable isotopes (mass units from Iso-1.1 to Iso-1.5, Iso-1.7, and Iso-1.8; see Supplementary Table S1.1 for the isotopic amino acids); the remaining four PC-MTs were also comprised of the core sequence of mass unit 1 but extended by 1–3 glycine and serine amino acids on the termini (mass units 1.2–1.5), which was not expected to significantly alter the MALDI-MS ionization efficiency. To demonstrate similar ionization efficiencies, equimolar mixtures of these isotopic and extended PC-MTs were prepared, photocleaved, and analyzed by standard nonimaging MALDI-MS, with peak intensities across all yielding a % CV of <20%.

**Figure 3:**
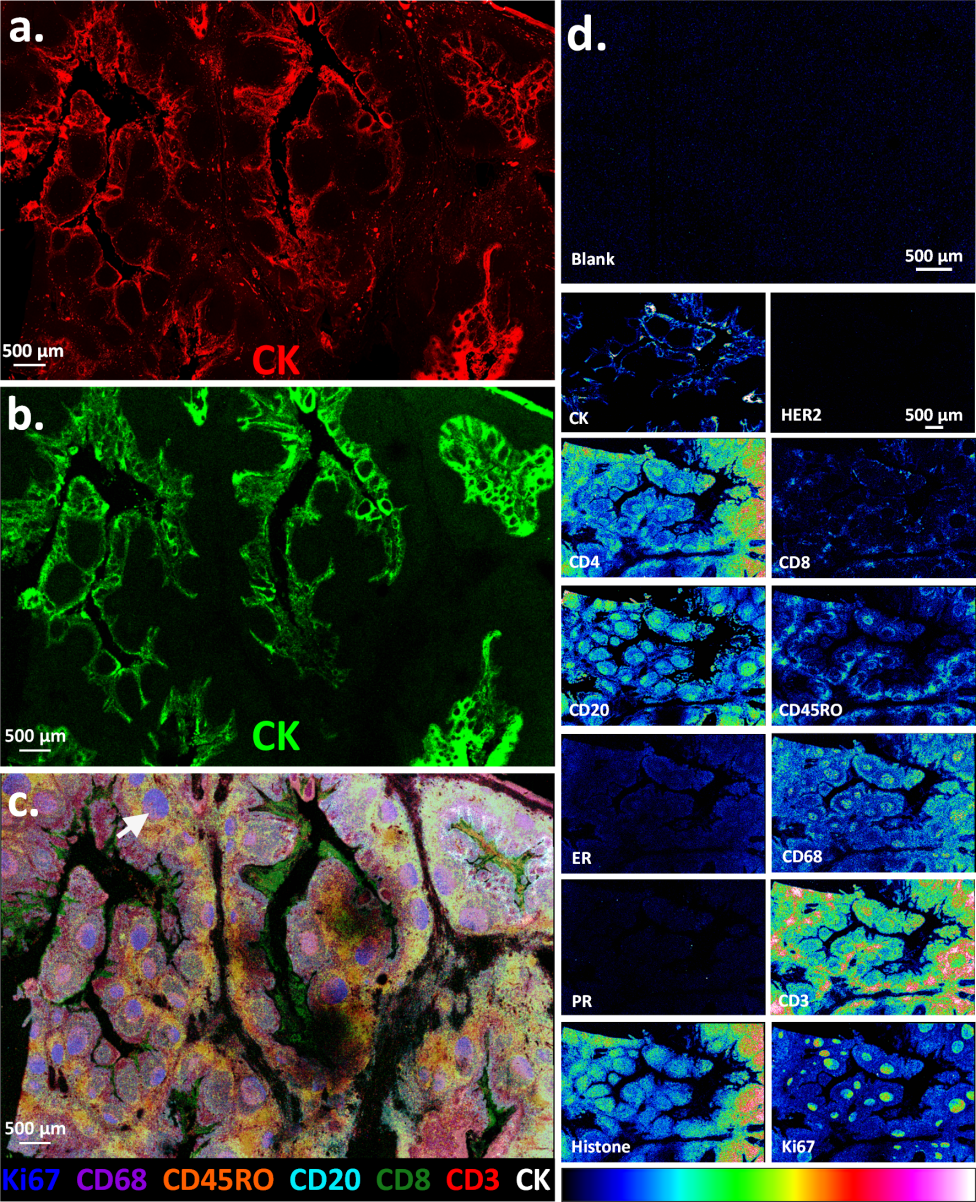
Multiplex MALDI mass spectrometry-based immunohistochemistry (MALDI-IHC) on 12 biomarkers in human tonsil FFPE tissue sections. MALDI-IHC was performed as in Figure 2 except 12 different biomarkers were used (see Supplementary Table S1 for PC-MT assignments for each antibody). Furthermore, the pan-cytokeratin antibody (CK) was labeled with both a PC-MT and fluorophore. (a) CK immunofluorescence image of the whole tonsil tissue section taken at a 5 μm resolution using a GenePix 4200A fluorescence microarray scanner. (b) MALDI-MS image of the same tissue section showing an intensity map of the monoisotopic *m*/*z* value for the PC-MT from the CK antibody (all MALDI-MS images have a 10 μm spatial resolution). The display scale (arbitrary peak intensity units) is as follows (minimum intensity/full intensity threshold): 5/50 (CK). (c) Multicolor MALDI-MS image overlay of selected biomarkers from the whole-tissue section, which show differential structural patterns. The display scale (arbitrary peak intensity units), is as follows (minimum intensity/full intensity threshold): 3/25 (Ki67), 3/40 (CD68), 3/7 (CD45RO), 2/20 (CD20), 2/5 (CD8), 3/7 (CD3), and 5/50 (CK). Color coding is indicated in the key underneath the image. (d) Individual MALDI-MS images of all 12 biomarkers shown as a color gradient for a representative subregion of the tissue section (biomarker identities are indicated by the labels). The “blank” is also shown, which is an adjacent tissue section stained with an isotype-control IgG bearing a PC-MT (same PC-MT as CD3). The gradient color scale is shown at the bottom. For comparison, the display scale of all biomarkers is set to a full-intensity threshold of 25 (arbitrary peak intensity units) and a minimum display intensity of 2.5 except for CK, CD20, and Ki67, which produced exceptionally strong signals and were therefore set to 50 and 5, respectively.

To validate most of the antibodies in the 12-plex panel, we initially tested it on FFPE tonsil tissue specimens. Tonsil is frequently used as a positive control for immune cell CD markers,^50,51^ including for B-cells^51^ and T-cells,^52,53^ and is also known to be strongly positive for Ki67.^54^ Notably, the CK antibody used in the 12-plex antibody panel was dual-labeled with a PC-MT and the DyLight 650 fluorophore. This ability allows both fluorescence and MS images to be obtained from the same specimen and coregistered, and also assists with antibody validation. Figure 3a shows the CK immunofluorescence image of the whole-tissue section (5 μm image resolution using a GenePix 4200A microarray scanner). The CK antibody selectively stains the squamous epithelial layer that covers the tonsil and lines its many invaginations and crypts, as expected.^55^Figure 3b shows the corresponding MALDI-MS image of the CK PC-MT on the same tissue section, producing an identical pattern (10 μm resolution).

Figure 3c shows a multicolor MALDI-MS image corresponding to PC-MTs from seven selected biomarkers. The image indicates a clear differential distribution of the various biomarkers. Of note, the germinal centers (e.g., white arrow) within the lymphoid follicles are strongly positive for Ki67 (proliferation marker; blue in Figure 3c) and CD20 (B-cell marker; cyan in Figure 3c, see also CD20 in Figure 3d for a better visualization of this biomarker in the germinal centers). This is expected since the germinal centers are the sites known to contain proliferating B-cells.^56^ The strong Ki67 staining of the germinal centers also agrees with previously reported results using standard IHC.^54^ In contrast, the T-cells (e.g., CD3 and CD45RO, red and orange, respectively, in Figure 3c) are prevalent in the extra-follicular regions (as well as some detection in the follicles), which is also in agreement with previous reports.^53,57^ Interestingly, the CD8+ cytotoxic T-cells were not widely distributed in the tissue but were instead found in high concentrations in discrete zones within the tonsillar crypts in the peri- and intraepithelial zones (green in Figure 3c). This is expected since the tonsillar crypts are known to harbor or entrap microbes and pathogens.^58,59^ See Supplementary Figure S2 for an example spectrum taken from a single pixel of the MALDI-MS image, which contains 9 of the 12 PC-MTs used, including signal-to-noise ratios of the mass reporter peaks.

Figure 3d displays all 12 antibodies from this multiplex experiment as separate MS images on a representative subregion of the tissue section using a gradient color scale. The “blank” corresponds to a PC-MT-labeled isotype control IgG, which was used to probe a separate but adjacent tissue section (the same PC-MT as that on the CD3 antibody), and provides no detectable signal. In contrast, all the CD antibodies are positive to varying degrees, as are the CK, Ki67, and histone antibodies, as expected, with many showing different distribution patterns. Notably, HER2 and PR are negative, as expected, and while ER shows a slight positivity; this is not unexpected based on previous reports using standard IHC,^60^ which detected ER but not PR in tonsils (note that the tonsils here are of female origin).

**Figure 4:**
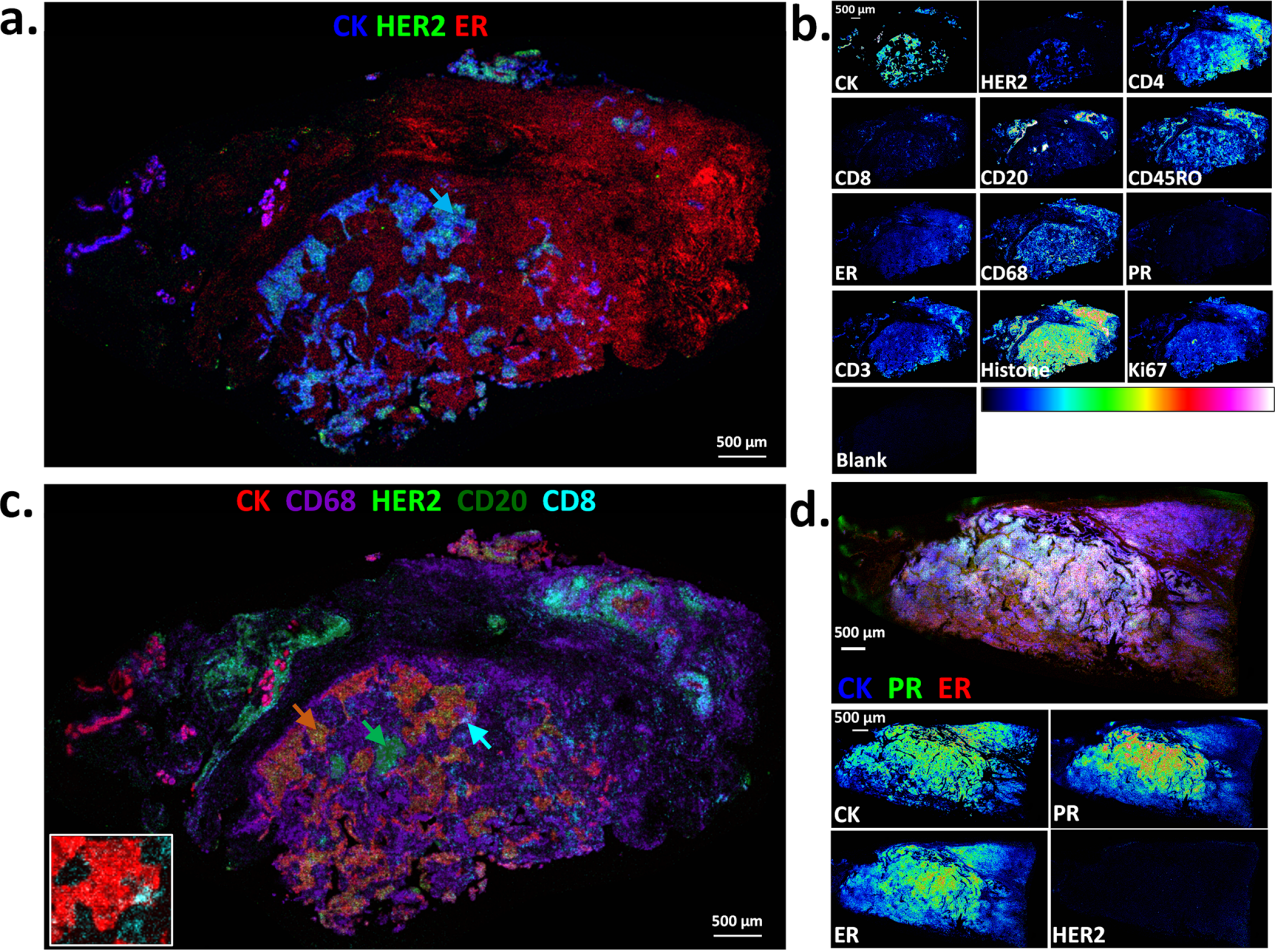
Multiplex MALDI mass spectrometry-based immunohistochemistry (MALDI-IHC) on 12 biomarkers in human breast cancer FFPE tissue sections. 12-Plex MALDI-IHC was performed as in Figure 3. The color coding of the multicolor images is indicated in the key for each, and the display scales listed below are in arbitrary peak intensity units (as the minimum intensity/full intensity threshold). (a) Multicolor MALDI-MS image overlay of a breast cancer tissue section showing an intensity map of the monoisotopic *m*/*z* values for the PC-MTs from CK, HER2, and ER (all MALDI-MS images have a 10 μm spatial resolution). The display scale is as follows: 1/35 (CK), 2/20 (HER2), and 2/8 (ER). According to traditional IHC, this tissue is PR–/ER–/HER2+ (see the main text for more detail). The blue arrow indicates an example tumor region. (b) Individual MALDI-MS images of all 12 biomarkers shown as a color gradient for the entire tissue section. The “blank” is also shown, which is an adjacent tissue section stained with an isotype-control IgG bearing a PC-MT (same PC-MT as CD3). The gradient color scale is also shown. For comparison, the display scale of all biomarkers is 2/20 except for CK, CD3, CD4, CD68, and Ki67, which produced exceptionally strong signals and are therefore set to 5/50. (c.) Multicolor MALDI-MS image overlay of the same breast cancer tissue section showing the selected biomarkers, which highlight tumor-infiltrating immune cells. The display scale is as follows: 1/30 (CK), 3/20 (CD68), 1/10 (HER2), 2/12 (CD20), and 2/7 (CD8). The orange, green, and cyan arrows indicate the tumor (colocalized CK and HER2), CD20+ B-cells, and CD8+ cytotoxic T-cells, respectively. The inset image on the lower left is a magnification of the region indicated by the cyan arrow, showing only CK and CD8. (d.) Multicolor MALDI-MS image overlay (top panel) and individual gradient-scale MALDI-MS images (bottom panels) of selected biomarkers on a different breast cancer tissue section. In this case, this tissue is PR+/ER+/HER2– according to traditional IHC (see the main text for more detail). The display scale for the multicolor image is as follows: 6/55 (CK), 2/20 (PR), and 2/15 (ER). For comparison, the display scale of the gradient color images is 2/20 except for CK, which produced an exceptionally strong signal and is therefore set to 10/100.

To further evaluate MALDI-IHC, the same 12-plex antibody panel was applied to breast cancer FFPE tissue specimens. The specimens were obtained from OriGene Technologies, Inc. (Rockville, MD) along with clinical annotations based on a pathologist review of traditional IHC and hematoxylin and eosin staining. MALDI-IHC results for one breast cancer sample are shown in Figure 4a–c. This specimen was classified based on the provided annotations as adenocarcinoma of breast (ductal), TNM staging of pT1 cPN3apMX, minimum stage-grouping IIIC, 75% tumor, and PR–/ER–/HER2+. First, to illustrate correlation of the MALDI-IHC results with the clinical annotations, Figure 4a shows a multicolor overlay of only 3 of the 12 biomarkers (CK, HER2, and ER) for simplicity, which are denoted with the primary colors. The tumor (e.g., the blue arrow) is indicated by the colocalized CK (blue, epithelial marker) and HER2 (green). The positive HER2 detection in the tumor agrees with the clinical annotations (PR–/ER–/HER2+). PR is negative throughout the tissue section, which is also in agreement with the clinical annotations (not shown in Figure 4a; see Figure 4b for all 12 biomarkers shown individually as well as the blank). While ER is moderately positive (red in Figure 4a), it is restricted to the extra-tumoral regions of the tissue section, which in agreement with the clinical annotations indicating the tumor itself is negative for ER.

Regarding the infiltrating immune cells, Figure 4c shows a five-color MALDI-MS image overlay of some examples from the same tissue section. In this case, CK is colorized red and HER2 as light green to indicate the tumor, with the colocalization of the two colors often appearing yellow-orange (e.g., the orange arrow). Discrete and dense patches of CD20+ B-cells (dark green) were observed in the intertumoral regions (e.g., the green arrow). The CD8+ cytotoxic T-cells (cyan) were again not widely distributed across the tissue, as with the tonsil tissue, but strong signals were observed in discrete zones, including adjacent to and infiltrated into the tumor (e.g., the cyan arrow of Figure 4c; see also the magnified inset image of this region where only CK and CD8 are colorized). The prevalence of CD8+ T-cells and their infiltration into the tumor have been reported as positive prognostic indicators for some forms of breast cancer, such as triple-negative breast cancer.^61−63^ There is also a highly abundant CD68 staining (purple) in the intertumoral regions, which is indicative of macrophages (and other mononuclear phagocytes).^46^ The abundant CD68 staining is consistent with reports that macrophages can often comprise up to 50% of the tumor mass.^47^ The presence of tumor-associated macrophages (TAMs) can indicate a positive or negative prognosis for a variety of solid tumors, although it is usually negative due to their tumor-promoting activities, including immunosuppression and the promotion of angiogenesis and inflammation.^47,64^

Finally, to further validate the PR, ER, and HER2 antibodies, a second breast cancer tissue specimen was analyzed. In this case, the clinical annotations according to the pathology report provided by the biospecimen vendor (OriGene) were as follows: adenocarcinoma of breast, ductal, lobular, metastatic, TNM staging of T2N2aMX, minimum stage-grouping IIIA, 95% tumor, and PR+/ER+/HER2– according to traditional IHC (i.e., the PR/ER/HER2 profile is the inverse of the previous tissue). The MALDI-MS image in the top panel of Figure 4d again shows a three-color image overlay using the primary colors for simple visualization in this case of PR, ER, and CK. PR (green) and ER (red) are both strongly positive, and colocalization with the CK epithelial biomarker (blue) produces a white color in many areas (occurring when all 3 colors are of a similar intensity). Figure 4d (lower panels) also shows CK, PR, ER, and HER2 separately, again with each as a gradient color, indicating PR+/ER+/HER2– in full agreement with the pathology report.

### Untargeted Label-Free and Targeted MALDI-MSI on the Same Tissue Section

**Figure 5:**
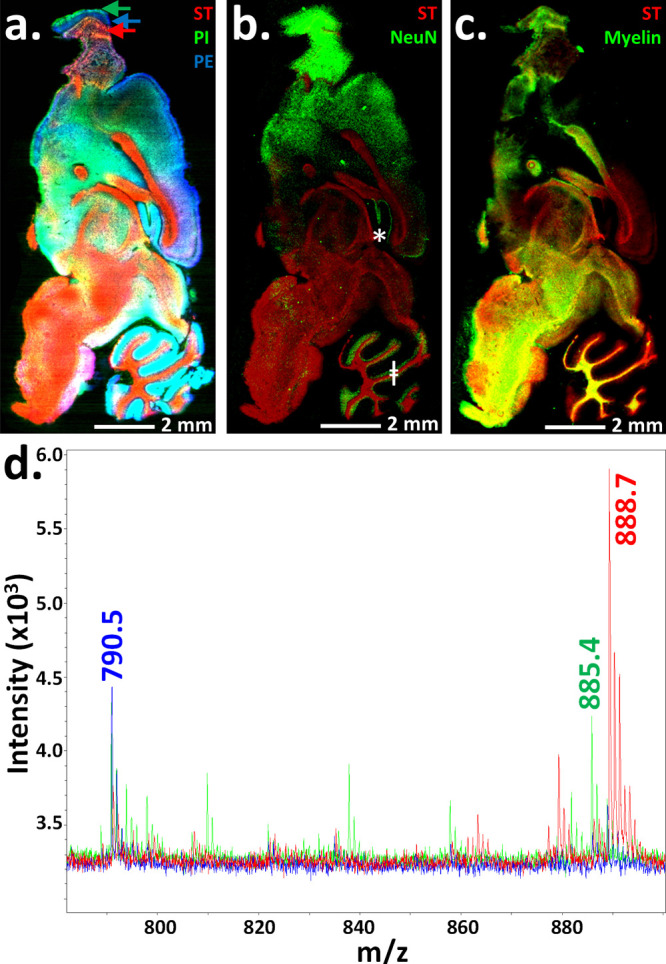
Untargeted small-molecule MALDI-MSI and targeted MALDI-IHC on the same tissue section. (a) Direct untargeted MALDI-MSI was first performed on an unfixed fresh-frozen mouse brain sagittal tissue section (all MALDI-MS images have a 20 μm spatial resolution). Three well-known lipid species (sulfatide (ST), *m*/*z* 888.7; phosphatidylinositol (PI), *m*/*z* 885.4; and phosphatidylethanolamine (PE), *m*/*z* 790.5) are shown in the colorized MALDI-MS image (red, green, and blue, respectively). The display scale (arbitrary peak intensity units) is as follows (minimum intensity/full intensity threshold): 5/14 (ST), 6/8 (PI), and 7/10 (PE). (b and c) The same tissue section was then processed for a second round of MALDI-MSI. To do so, the matrix was washed away, the tissue was fixed, and MALDI-IHC was performed to detect macromolecular antigens using PC-MT-antibodies. For demonstration purposes, images of selected biomolecules from the first and second rounds of MALDI-MSI were overlaid. (b) Sulfatide (red) from the first round of MALDI-MSI (direct small-molecule detection) is overlaid with the image of NeuN (green) from the second round of MALDI-MSI (MALDI-IHC). The display scale (arbitrary peak intensity units) is as follows (minimum intensity/full intensity threshold): 8/20 (ST) and 1/5 (NeuN). (c) Sulfatide (red) from the first round of MALDI-MSI (direct small-molecule detection) is overlaid with the image of myelin basic protein (green) from the second round of MALDI-MSI (MALDI-IHC). The display scale (arbitrary peak intensity units) is as follows (minimum intensity/full intensity threshold): 8/20 (ST) and 2/20 (myelin). (d) Example overlaid spectra from the first round of MALDI-MSI (direct small-molecule detection) are shown, which were color-coded to match the image in panel a. (the three lipid masses are indicated). The color-coded arrows in panel a indicate the regions from which the MALDI-MS spectra were derived.

It would be highly advantageous to detect both untargeted label-free small molecules and macromolecules targeted by MALDI-IHC on the same tissue section. For example, this would allow the colocalization of small-molecule drugs and drug-targets, such as their receptors, as well as associated biomolecules involved in the cellular response to the drug. To demonstrate the basic feasibility, we first performed a direct MALDI-MSI analysis on fresh-frozen (FF) mouse brain sagittal tissue sections (FF is optimal instead of FFPE to facilitate small-molecule detection without tissue fixation or prior washing). The negative ion mode MALDI-MSI with the DAN matrix was used. Next, the tissue was washed and fixed with cold acetone to remove the MALDI-MS matrix compound, then fixed further with paraformaldehyde. MALDI-IHC was then performed as described earlier, constituting a second round of MALDI-MSI. The result of the first round of direct MALDI-MSI is shown in Figure 5a, with the image color-coded for the *m*/*z* values of three well-known lipids identified from the METLIN^65^ database of the Scripps Center for Metabolomics and in agreement with the prior MALDI-MSI analysis of lipids from mouse brain tissue sections^66^ (sulfatide (24:1), red, *m*/*z* 888.7; phosphatidylethanolamine (40:6), blue, *m*/*z* 790.5; and phosphatidylinositol (38:4), green, *m*/*z* 885.4). These three lipids are clearly enriched in different structures of the brain; in particular, sulfatide (red) shows a distinct pattern from the other two lipids. However, there is also a significant colocalization of phosphatidylethanolamine (blue) and phosphatidylinositol (green), as would be expected as two of the major structural lipids of eukaryotic cellular membranes^67^ (colocalized blue and green appears as cyan in Figure 5a). For demonstration purposes, Figure 5b and Figure 5c show two-color overlays of the sulfatide lipid, which was detected in the first round of direct MALDI-MSI, with selected macromolecular biomarkers detected by MALDI-IHC in the second round of MALDI-MSI. Figure 5b shows sulfatide (red) overlaid with the neuronal *nuclear* biomarker NeuN (green) that was detected by MALDI-IHC. These two biomolecules generally do not colocalize. Conversely, Figure 5c shows the same lipid, sulfatide (red), overlaid with myelin basic protein (green) that was detected by MALDI-IHC. In this case, there is a strong colocalization of sulfatide and myelin (as evidenced by the yellow occurring from the colocalization of the green and red colors). The colocalization of sulfatide and myelin agrees with previous literature, which indicates sulfatide is predominantly found in the myelin sheath (Schwann cells or oligodendrocytes) of neuronal axons.^68,69^ Conversely, sulfatide would not be expected to colocalize with the neuronal *nuclear* biomarker NeuN as was observed here (note that, as shown earlier in Figure 2, myelin and NeuN also generally do not colocalize; also note that the myelin and NeuN imaging in Figure 5 is highly similar to that obtained previously with FFPE tissues in Figure 2, indicating that this dual-imaging procedure does not promote target-protein delocalization). Finally, example spectra from the first round of direct MALDI-MSI are shown in Figure 5d. These spectra are from selected image pixels chosen from the three different “layers” observed in the olfactory bulb and nerve region of the mouse brain (see the colored arrows in Figure 5a for these “layers”). In the future, the utilization of MS/MS or higher resolution FTICR mass spectrometers will allow for more accurate small-molecule identification.

## Conclusions

The goal of this work was to further develop the ability of MALDI-MSI to perform highly multiplexed imaging of targeted biomolecules in biospecimens. Such a capability would provide a major tool for systems biologists who require a detailed knowledge of the distribution of key biomolecules in complex tissues at the cellular and multi-cellular levels covering areas significantly larger (e.g., >1 cm^2^) than can be normally covered by subcellular imaging mass cytometry. It would also provide pathologists with a powerful new tool to analyze tumor-tissue specimens to ultimately obtain improved therapy and patient outcomes. In an approach termed MALDI-IHC, we have demonstrated the ability to simultaneously image potentially hundreds of targeted biomarkers in FFPE and FF thin-tissue sections from the mouse brain as well as from human tonsil and breast cancer at a 10 μm spatial resolution using novel PC-MTs, which were incorporated into antibody probes in a one-step reaction. In contrast, conventional light microscopy-based IHC methods can image only a few targeted biomolecules. Enhancements of MALDI-IHC also demonstrated here include the following: (i) the development of dual-labeled PC-MT and fluorescent antibody probes that enable the use of both MSI and fluorescence imaging on the same specimen with the same probes and (ii) mapping the spatial distribution and colocalization of both unlabeled small molecules, such as phospholipids, and PC-MT-antibody-labeled biomolecules, such as large protein antigens, on the same tissue section over >1 cm^2^ regions. These advances will potentially provide more powerful methods for researchers to explore the spatial distribution of biomolecules in tissues at the cellular level in various fields, including proteomics, tissue pathology, tissue diagnostics, therapeutics, and precision medicine.

## Supporting Information Available

Materials and methods for probing streptavidin beads with PC-MT-abs, bead-array formation, MALDI-IHC, and immunofluorescence staining (PDF)
Mass unit sequences for PC-MTs and mass reporter masses, amino acid isotopes, demonstration of 15-plex MALDI-MSI on bead-arrays as a model system using PC-MT-abs, and example spectrum and PC-MT signal-to-noise ratios from the MALDI-IHC analysis of human tonsil tissues (PDF)

## Abbreviations

DAN 1: 5-diaminonaphthalene
DHB 2: 5-dihydroxybenzoic acid
DESI: desorption electrospray ionization
ESI: electrospray ionization
FFPE: formalin-fixed paraffin-embedded
FF: fresh-frozen
IHC: immunohistochemistry
ITO: indium tin oxide
ISH: *in situ* hybridization
LDI: laser desorption/ionization
MSI: mass spectrometric imaging
MS: mass spectrometry
MS-Water: mass spectrometry-grade water
MALDI: matrix-assisted laser desorption/ionization
MALDI-IHC: multiplexed MALDI mass spectrometric imaging-based IHC
NHS: *N*-hydroxysuccinimide
PC: photocleavable
PC-BAMS: photocleavable bead-array mass spectrometry
PC-Biotin: photocleavable biotin
PC-Linker: photocleavable linker
PC-MT: photocleavable mass-tag
PC-MT-antibody: photocleavable mass-tag antibody
