# Supplementary material for: Highly Multiplexed Immunohistochemical MALDI-MS Imaging of Biomarkers in Tissues
Source: J Am Soc Mass Spectrom. 2021 Feb 25;32(4):977–88. doi: 10.1021/jasms.0c00473 (PMC8033562; doi:10.1021/jasms.0c00473)
Supplement: Supplementary file 1 — js0c00473_si_001.pdf [file js0c00473_si_001.pdf]

Materials

1,5-diaminonaphthalene (DAN, 97%), 2,5-dihydroxybenzoic acid (DHB, 98.0%), paraformaldehyde (powder, 95%), citrate buffer (pH 6.0, 10x) and octyl ß-D-glucopyranoside (OBG) (50% [w/v] stock solution) were from Sigma Aldrich (St. Louis, MO). Tween®-20, molecular biology grade was from Promega (Madison, WI). Antigen Retrieval Reagent-Basic (CTS013), Antigen Retrieval Reagent-Universal (CTS015) and Antigen Retrieval Reagent-Acidic (CTS015) were from R&D Systems/BioTechne (Minneapolis, MN). DyLight 650 NHS ester and anti-streptavidin antibody clone S3E11 were from Thermo Fisher Scientific (Waltham, MA). Gold coated microscope slides were from Angstrom Engineering, Inc. (Kitchener, ON, Canada). Mouse C57 brain sagittal FFPE sections (5 µm thickness) and mouse C57 brain sagittal Fresh Frozen (FF) sections (embedded in 2% w/v CMC, 10 µm thickness) were from Zyagen (San Diego, CA). Breast cancer tissue FFPE blocks were from OriGene Technologies, Inc. (Rockville, MD) and normal tonsil tissue FFPE blocks were from Amsbio LLC (Cambridge, MA), all were sectioned (5 µm thickness) and mounted onto slides by Zyagen (San Diego, CA). Mouse IgG whole molecule (015-000-003), normal mouse serum (015-222-001), rabbit IgG whole molecule (011-000-003) and normal rabbit serum (011-000-001) were from Jackson ImmunoResearch Laboratories, Inc. (West Grove, PA). Streptavidin-coated 20 µm PMMA beads (microspheres) were from PolyAn GmbH (Berlin, Germany). 0.5 mL Ultrafree-MC centrifugal 0.45 µm filter devices were from Millipore Sigma (Burlington, MA). PD SpinTrap G-25 columns were from GE Healthcare Life Sciences (Pittsburgh, PA). MALDI-IHC and bead-array antibodies were obtained from various suppliers as follows: human/mouse/rat anti-myelin basic protein (MAB42282) from R&D

Systems/BioTechne (Minneapolis, MN); anti-NeuN antibody, clone A60 (MAB377) and anti-synapsin-2 antibody, clone 19.4 (MABN1573) from Millipore Sigma (Burlington, MA); human/mouse anti-GLUT1 (PA146152) from Fisher Scientific (Hampton, NH); and anti-MAP2 antibody [AP-20] (ab11268), recombinant anti-NeuN (ab209898; BSA and azide free), recombinant anti-myelin basic protein (ab230378; BSA and azide free), recombinant rabbit IgG, monoclonal [SP137] - isotype control (ab208334; BSA and azide free), recombinant anti-pan cytokeratin antibody [C-11] (ab264485; BSA and azide free), recombinant anti-CD3 epsilon antibody [CAL57] (ab251607; BSA and azide free), recombinant anti-CD4 antibody [EPR6855] (ab181724; BSA and azide free), recombinant anti-CD8 alpha antibody [CAL66] (ab251596; BSA and azide free), recombinant anti-CD20 antibody [EP459Y] (ab214282; BSA and azide free), anti-CD45RO antibody [UCH-L1] (ab23), recombinant anti-estrogen receptor alpha antibody [SP1] (ab187260; BSA and azide free), recombinant anti-progesterone receptor antibody [YR85] (ab206926; BSA and azide free), recombinant anti-ErbB 2 antibody [CAL27] (ab251602; BSA and azide free), recombinant anti-histone H2A.X antibody [EPR22820-23] - ChIP (ab256544; BSA and azide free), recombinant anti-CD68 antibody [EPR20545] (ab227458; BSA and azide free), and recombinant anti-Ki67 antibody [EPR3610] (ab209897; BSA and azide free) were from Abcam (Cambridge, MA). FlexWell™ 16-Chamber Self-Adhesive Gaskets (204916) were from Grace Bio-Labs (Bend, Oregon).

Methods

Handling of photocleavable reagents. Photocleavable reagents were protected from light during all long incubation steps (≥ 15 min) and during storage, but were otherwise handled under ambient laboratory lighting.

Probing streptavidin beads with PC-MT-antibodies and bead-array formation. Streptavidin-coated 20 µm PMMA beads were processed in 0.5 mL Ultrafree-MC centrifugal 0.45 µm filter devices unless otherwise noted (washing was performed by 3 s vortex mixing of bead suspensions in the filter devices and filtration was performed for 5 s at 15,000 rpm on a standard micro-centrifuge). For each PC-MT-antibody version, 10,000 beads were used and processed separately unless otherwise noted. Beads were first washed 4x 400 µL with Bead Block Buffer (1% BSA [w/v] in TBS-T; note TBS-T is TBS supplemented with 0.05% [v/v] Tween-20). Beads were then probed separately with 15 different versions of an anti-streptavidin PC-MT-antibody, each PC-MT-antibody version carrying a different PC-MT species (*i.e.* different mass unit; see Supplementary Table S1 for mass units and Supplementary Table S1.1 for stable isotopic amino acids used in some mass units). PC-MT-antibodies were diluted to 1 µg/mL in Bead Block Buffer and probing was performed using 100 µL for 1 hr with gentle mixing. Beads were then washed 4x 400 µL with TBS-T followed by pooling all 15 different bead versions. Pooled beads were then washed further 4x 400 µL with mass spectrometry grade water (MS-Water). Formation of random bead-arrays on indium tin oxide (ITO) coated microwell substrates having the footprint of a standard microscope slide was performed as

previously reported [1, 2] (note that after bead-array formation slides were dried 45 min in a vacuum desiccation chamber).

Multiplex mass spectrometry-based immunohistochemistry (MALDI-IHC). For deparaffinization and hydration, FFPE tissue sections were treated as follows (each treatment step in separate staining jars): 3x with xylene for 5 min each; 1x with xylene:ethanol (1:1) for 3 min; and then hydrate 2x with 100% ethanol for 2 min each, 1x with 95% ethanol for 3 min each, 1x with 70% ethanol for 3 min, 1x with 50% ethanol for 3 min and 1x with TBS for 10 min. Antigen retrieval was achieved in 200 mL of 95°C 1X citrate buffer (pH 6.0; see Materials) for 1 hr and followed by cooling in the same beaker for 30 min at room temperature. Alternatively, acidic or basic antigen retrieval was achieved for tonsil and breast cancer tissues in 60 mL of 95°C 1X Antigen Retrieval Reagent-Acidic or 1X Antigen Retrieval Reagent-Basic (see Materials) for 30 min and followed by cooling in the same Coplin staining jar for 30 min at room temperature. Slides were then blocked in a staining jar for 1 hr with 50 mL Tissue Blocking Buffer (2% [v/v] normal serum [rabbit and mouse] and 5% (w/v) BSA in TBS-T). For PC-MT-antibody staining, slides were treated at 4°C for overnight with 200 µL/section of a solution containing 2.5 µg/mL of each antibody for mouse brain and 0.5 µg/mL of each antibody for human tonsil and breast cancer, diluted in Tissue Blocking Buffer (incubation was performed protected from light, in a humidified chamber to avoid evaporation and with each tissue section surrounded by hydrophobic barrier pen to retain the fluid). The slides were next washed as follows: 3x 5 min each with TBS followed by 3x 2 min each with 50 mM ammonium bicarbonate (note, all solutions were in MS-Water and all washes were

performed using excess solution with the slides placed horizontally in a petri dish with gentle shaking). Tissue slides were ultimately dried for 45 min in a vacuum desiccation chamber.

Immunofluorescence staining. In some cases, immunofluorescence was performed instead of staining with PC-MT-antibodies. In these cases, antibodies were labeled with a 15-fold molar excess of a DyLight 650 NHS Ester reagent (added from a 5 mM stock in DMF). Immunostaining with fluorescent antibodies was otherwise performed in the same manner as with the PC-MT-antibodies. Fluorescence imaging of dried slides was performed on a GenePix 4200A fluorescence scanner at 5 µm resolution (Molecular Devices, San Jose, CA).

References

1. Zhou, Y., Liu, Z., Rothschild, K.J., **Lim, M.J.**: Proteome-wide drug screening using mass spectrometric imaging of bead-arrays. Sci Rep. **6**, 26125 (2016)
2. Lim, M.J., Liu, Z., Braunschweiger, K.I., Awad, A., Rothschild, K.J.: Correlated matrix-assisted laser desorption/ionization mass spectrometry and fluorescent imaging of photocleavable peptide-coded random bead-arrays. Rapid Commun Mass Spectrom. **28**, 49-62 (2014)
