# Supplementary material for: Highly Multiplexed Immunohistochemical MALDI-MS Imaging of Biomarkers in Tissues
Source: J Am Soc Mass Spectrom. 2021 Feb 25;32(4):977–88. doi: 10.1021/jasms.0c00473 (PMC8033562; doi:10.1021/jasms.0c00473)
Supplement: Supplementary file 2 — js0c00473_si_002.pdf [file js0c00473_si_002.pdf]

Supplementary Table S1. Mass Unit Sequences for PC-MTs and Mass Reporter Masses

Mass Unit ID	Mass Unit Amino Acid Sequence*	Mass Reporter Mass (Monoisotopic)*	Antibody
1	APRLRFYSL	1,206.72	Myelin, CK, Streptavidin
Iso-1.1	(A)PRLRFYSL	1,210.74	CD3
Iso-1.2	(AP)RLRFYSL	1,216.75	CD4
Iso-1.3	A(P)R(L)RFYSL	1,222.79	CD8
Iso-1.4	(AP)R(L)RFYSL	1,226.82	CD20
Iso-1.5	A(P)R(L)R(F)YSL	1,230.84	CD45RO
Iso-1.6	(AP)R(L)R(F)YSL	1,234.87	-
Iso-1.7	A(P)R(L)R(F)YS(L)	1,240.91	ER
Iso-1.8	(AP)R(L)R(F)YS(L)	1,244.93	PR
1.1	APRLRFYSLG	1,263.74	-
1.2	SAPRLRFYSL	1,293.75	HER2
1.3	GAPRLRFYSLG	1,320.76	Histone H2A.X
1.4	SAPRLRFYSLG	1,350.76	CD68
1.5	GAPRLRFYSLGG	1,377.78	Ki67
2	RPPGFSFFR	1,194.67	NeuN, Streptavidin
3	IPSINVHHY	1,163.65	Streptavidin
4	YHWYGYTPQNVI	1,624.80	GLUT1
5	APLFYSL	894.52	Synapsin-2, Streptavidin
6	PPGFSFF	882.46	MAP2, Streptavidin
7	LRRASLG	856.56	Streptavidin
8	PPGASPFR	912.52	Streptavidin
9	RYPFPGP	917.51	Streptavidin
10	SFLLRNP	930.57	Streptavidin
11	PPGPSPFR	938.53	Streptavidin
12	RPPGFSPL	954.57	Streptavidin
13	PPGESPFR	970.52	Streptavidin
14	PPGFSPFR	988.55	Streptavidin
15	RGYGYQGL	997.54	Streptavidin
16	RGYAYQGL	1,011.55	Streptavidin

Light blue shaded rows are mass unit 1 or variations of mass unit 1 used to minimize variable MALDI-MS ionization efficiency. The red and green letters represent stable isotopic amino acids shown in Supplementary Table S1.1.

*Mass units are N-terminal acetylated on the α-amine; the mass reporter masses include this acetylation plus the mass unit and a small portion of the photocleaved PC-Linker (see Fig. 1 Step 3).

Supplementary Table S1.1. Amino Acid Isotopes

1-Letter	Isotopic Amino Acid	FMOC-Isotopic Amino Acid
A	L-ALANINE (2,3,3,3-D4, 98%)	L-ALANINE-N-FMOC (2,3,3,3-D4, 98%)
P	L-PROLINE (13C5, 99%; 15N, 99%)	L-PROLINE-N-FMOC (13C5, 99%; 15N, 99%)
L	L-LEUCINE (D10, 98%)	L-LEUCINE-N-FMOC (D10, 98%)
F	L-PHENYLALANINE (D8, 98%)	L-PHENYLALANINE-N-FMOC (D8, 98%)

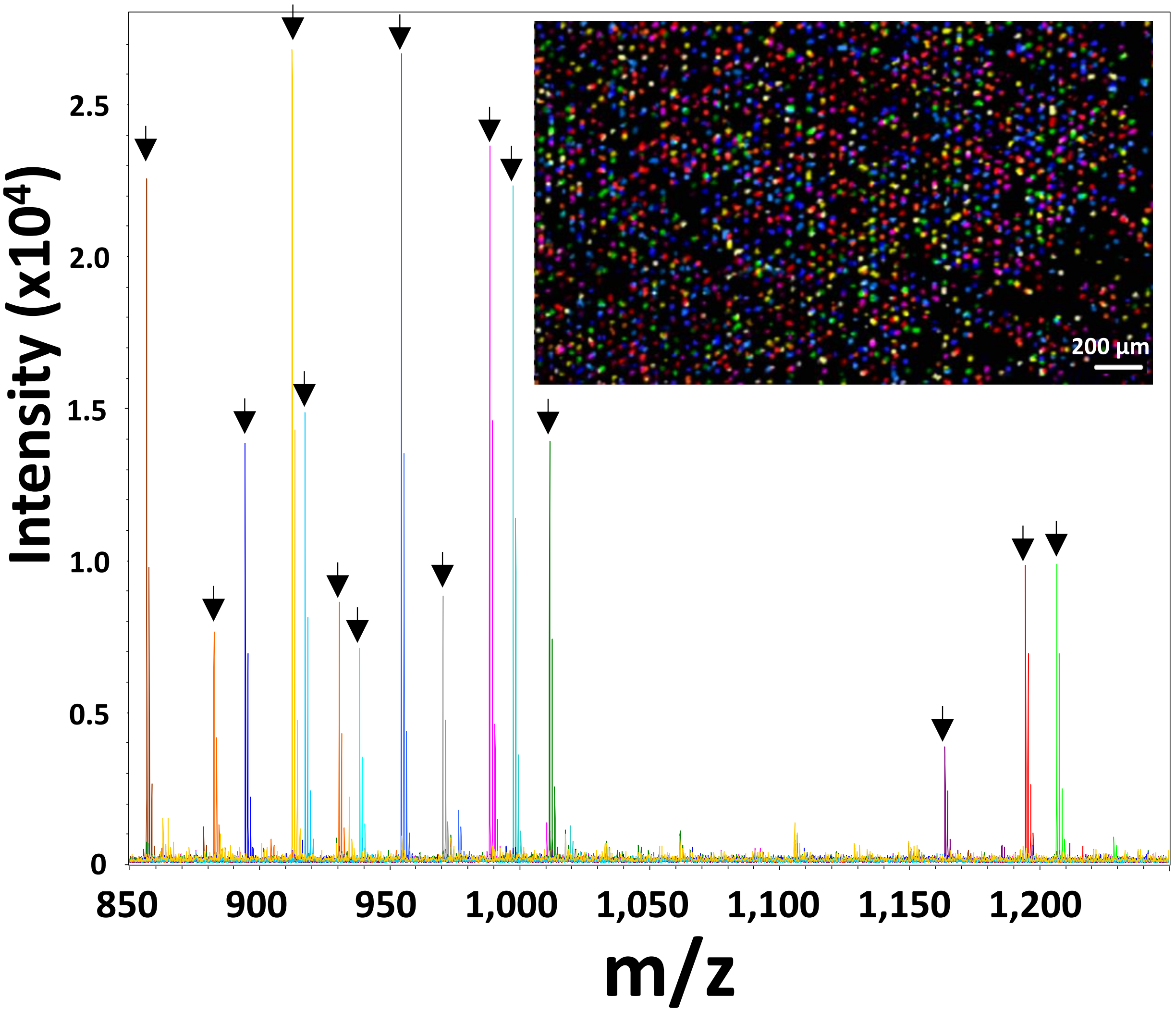

Supplementary Figure S1. Demonstration of 15-plex MALDI-MSI on bead-arrays as a model system using PC-MT-antibodies. 15 different anti-streptavidin PC-MT-antibodies were created by direct 1-step labeling with different PC-MTs. Each PC-MT-antibody species was used in a separate reaction to probe 20 µm polymer streptavidin beads. The resultant 15 bead species were then pooled and used to form an array on a substrate having the footprint of a microscope slide, which was then subjected to MALDI-MSI. A colorized 15-plex MALDI-MS image of the bead-array is shown corresponding to the 15 different monoisotopic mass reporter m/z values from the various PC-MT-antibodies. The color-coded overlaid spectra provided are from representative single pixels from within single beads for each of the 15 different bead species (black arrows indicate the mass reporters). Note that while 1 Da separated natural isotopes of the mass reporters are easily resolved by the MALDI-MS, they are not discernable in the spectra provided due to the compact X-axis scaling. MALDI-MS image is 10 µm spatial resolution.

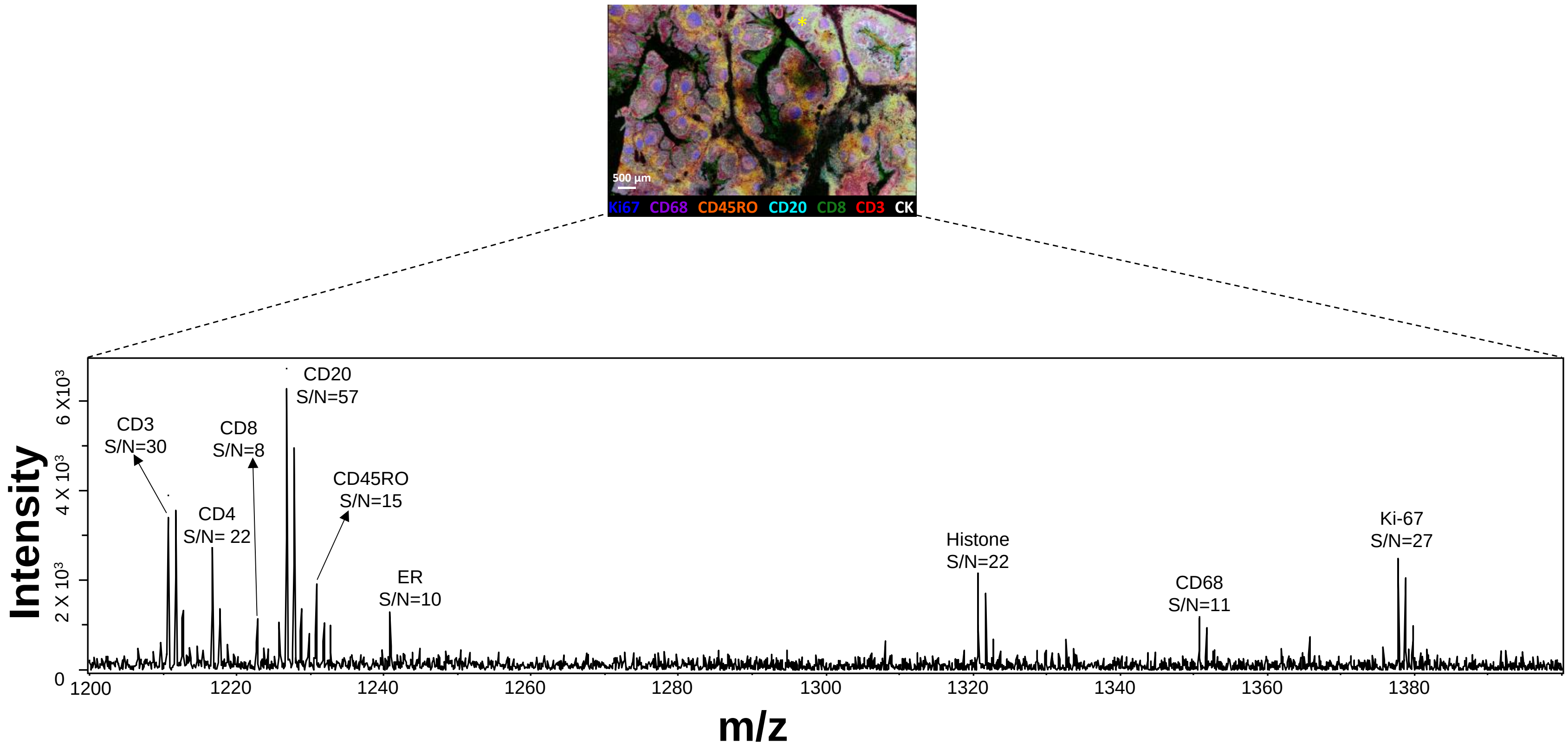

Supplementary Figure S2. Example spectrum and PC-MT signal-to-noise ratios from MALDI-IHC analysis of human tonsil tissue. The MALDI-MS image shown is that from Fig. 3c of the main manuscript. The yellow asterisk indicates the region of the image from which the spectrum in the lower panel was acquired (from a single pixel), which contains 9 of the 12 PC-MTs used. Each monoisotopic PC-MT reporter peak in the spectrum is labeled with the corresponding antibody and signal-to-noise ("S/N") ratio.
